# Minimal Disease Activity as A New Therapeutic Target in Atopic Dermatitis: A 5‐Year Real‐Life Experience With Dupilumab

**DOI:** 10.1111/ijd.70260

**Published:** 2026-01-13

**Authors:** Francesco Leo, Luca Mastorino, Luca Cangialosi, Davide Favre, Chiara Anna Fiasconaro, Yingying Liao, Federico Goso, Niccolò Siliquini, Giovanni Cavaliere, Pietro Quaglino, Simone Ribero, Michela Ortoncelli

**Affiliations:** ^1^ Dermatology Clinic, Department of Medical Sciences University of Turin Turin Italy

**Keywords:** atopic dermatitis, Dupilumab, minimal disease activity, real‐life

## Abstract

**Background:**

Atopic dermatitis (AD) is a chronic inflammatory skin disease with significant physical and psychosocial burden. Dupilumab, a monoclonal antibody targeting IL‐4Rα, has proven to be effective for moderate‐to‐severe AD, but long‐term real‐world data remain limited.

**Objective:**

The aim of this study is to evaluate the long‐term effectiveness, safety, and achievement of minimal disease activity (MDA) in patients with moderate‐to‐severe AD treated with dupilumab over a 5‐year period.

**Methods:**

A retrospective single‐center cohort study was conducted at the Dermatologic Clinic of the University of Turin, including 583 patients aged ≥ 6 years treated with dupilumab between November 2018 and January 2025. Effectiveness was measured by Eczema Area and Severity Index (EASI), Pruritus‐Numerical Rating Scale (P‐NRS), and Sleep Loss‐Numerical Rating Scale (S‐NRS) scores, Dermatology Life Quality Index (DLQI). MDA was defined as EASI‐90 combined with P‐NRS ≤ 1. Safety and drug survival were also assessed.

**Results:**

Dupilumab treatment resulted in significant and sustained reductions in EASI and symptom scores over 5 years. The proportion of patients achieving MDA increased from 22.0% at 16 weeks to 56.0% at 208 weeks and remained stable at 55.3% through 5 years. Male sex and childhood onset may reduce treatment success, warranting further study. The safety profile was favorable, with low discontinuation rates, and conjunctivitis as the most common adverse event.

**Conclusion:**

Our long‐term real‐world data support the sustained effectiveness and safety of dupilumab in moderate‐to‐severe AD, with over 55% of patients achieving and maintaining MDA at 5 years.

## 1 Introduction

Atopic dermatitis (AD) is a common chronic inflammatory skin disease that affects both pediatric and adult patients, often representing the initial manifestation of the atopic march, followed by allergic rhinitis, food allergies, and asthma [1, 2, 3]. The psychosocial impact of moderate to severe AD is substantial, being associated with increased absenteeism, impaired productivity, reduced sleep quality, and elevated rates of depression and anxiety, significantly affecting overall quality of life [4, 5].

In recent years, various systemic therapies targeting the interleukin (IL), or Janus kinase‐signal transducer and activator of transcription (JAK–STAT) pathway have been approved for moderate‐to‐severe AD [ 3]. Among them, dupilumab is a monoclonal antibody that targets the interleukin‐4 receptor alpha (IL‐4Rα). Its mechanism of action involves the inhibition of interleukin‐4 (IL‐4) and interleukin‐13 (IL‐13) signaling, which are key cytokines implicated in diseases characterized by type II inflammation, such as ad [ 6].

Following the clinical trials that led to its initial approval for the treatment of moderate to severe AD in 2017 [7], dupilumab has demonstrated excellent effectiveness and safety profiles in numerous real‐life studies involving both adult and pediatric patients [8, 9]. Although real‐life data are increasingly available, evidence regarding long‐term outcomes remains limited.

The advent of novel systemic treatments has paved the way for more ambitious and clearly defined therapeutic goals, aimed at achieving substantial reductions in disease burden. Within this evolving framework, the concept of minimal disease activity (MDA) has gained prominence as an increasingly relevant standard for evaluating therapeutic response in AD [10, 11]. Similarly to long‐term data, studies related to the evaluation of achieving MDA are lacking.

In this monocentric study, we aim to analyze the effectiveness and safety of dupilumab in the treatment of moderate to severe AD in patients aged ≥ 6 years over a period of up to 5 years, with a focus on achieving and sustaining MDA over the long term, while assessing potential predictors of therapeutic response.

## 2 Methods

In this retrospective single‐center cohort study, we analyzed data from patients aged ≥ 6 years with a diagnosis of moderate‐to‐severe AD who were treated with dupilumab at the Dermatologic Clinic of the University of Turin (Italy) between November 2018 and January 2025, to evaluate its effectiveness and safety in a real‐life scenario.

Effectiveness has been assessed with the Eczema Area and Severity Index (EASI), evaluating its mean value, mean % reduction, mean % reduction and improvement of 75% or more (EASI‐75), mean % reduction and improvement of 90% or more (EASI‐90), and mean % reduction and improvement of 100% (EASI‐100) at 16, 52, 104, 156, 208, and 260 weeks of treatment. In addition to the overall EASI assessment, the Head and Neck EASI (H&N EASI) was also evaluated separately at each time point.

We also assessed the decline in the Pruritus‐Numerical Rating Scale (P‐NRS), Sleep Loss‐Numerical Rating Scale (S‐NRS), and Dermatology Life Quality Index (DLQI) over the course of the study.

MDA was defined as the simultaneous achievement of EASI‐90 and a P‐NRS score ≤ 1.

Moreover, the safety profile was appraised through an analysis of adverse effects observed during the treatment, as well as interruptions that occurred, with a specification of the corresponding reasons.

The dosage received by each patient was determined in accordance with weight and age parameters as specified in the technical data sheet. After a period of stable disease at the traditional dosage, some patients underwent a dose reduction, switching to administration intervals of 3 or 4 weeks.

Furthermore, the drug survival was assessed and reported after 5 years of treatment.

### 2.1 Statistical Analysis

Descriptive statistics were used to evaluate the characteristics according to the number of patients, mean values, and standard deviations for continuous variables and percentage values for categorical variables.

Categorical variables were analyzed using a chi‐square test or Fisher's exact test. About continuous variables, Student's t‐test or Mann–Whitney U test was used. Survival curves were evaluated by approximating using Kaplan–Meier curves and compared using log‐rank tests.

A statistical univariate analysis was conducted to evaluate the association between baseline variables and treatment outcomes over time. Variables considered in the model were gender, infantile onset of disease, family history of atopy, prior use of cyclosporine, baseline EASI score, and head/neck involvement. Furthermore, a multivariate logistic regression analysis was performed to evaluate the likelihood of achieving MDA, using the same baseline variables included in the univariate analysis.

Differences were considered statistically significant at *p* < 0.05.

## 3 Results

A total of 583 patients were included in the study. Baseline demographics, comorbidities, and prior therapies are summarized in Table 1. A total of 94 (16.3%) patients underwent treatment tapering, extending the dosing interval from every 2 weeks to every 3 or 4 weeks (mean time to tapering initiation was 28.6 months after treatment start).

**TABLE 1:** Baseline demographics, atopic comorbidities, clinical features, and previous therapies.

	Total (*N* = 583)
Sex *N* (%)	
Female	274 (47.0%)
Male	309 (53.0%)
Age at dupilumab start, years	
Mean (SD)	35.9 (18.8)
Median	31.0
Range	0–85
6–17 (%)	77 (13.2)
> 17	506 (86.8)
Duration of atopic dermatitis (SD)	21.6 (15.7)
Age at onset of AD, years	
Mean (SD)	34.5 (19.8)
Median	31.0
Range	0–85
Atopic comorbidities *N* (%)	
Allergic conjunctivitis	103 (17.8)
Food allergies	3 (0.5)
Asthma	122 (20.9)
Allergic rhinitis	40 (6.9)
Family history of AD	171 (29.3)
Family history of respiratory atopy	20 (3.4)
Clinical phenotype *N* (%)	
Flexural	501 (85.9)
Head‐and‐neck	449 (77.0)
Nummular	10 (1.7)
Prurigo nodularis	40 (6.9)
Generalized	421 (72.2)
Previous therapies *N* (%)	
Topical steroid	545 (93.5)
Systemic steroid	502 (85.1)
Topical calcineurin inhibitors	219 (37.6)
Cyclosporine	421 (72.2)
Narrowband UVB phototherapy	54 (9.3)
Tralokinumab	28 (4.8)
JAK inhibitors	3 (0.5)

Abbreviations: AD, atopic dermatitis; JAK, Janus kinase; UVB, ultraviolet B; SD, standard deviation.

EASI and patient reported outcome (PRO) scores showed a consistent reduction starting from the first follow‐up visit, scheduled after 16 weeks of treatment (Table 2). The lowest value ± SD was recorded at Week 208, reaching 1.4 ± 2.4 (Table 2). Comparable results were observed when specifically analyzing the EASI score for the head and neck region (H&N EASI). From the first follow‐up at Week 16, a marked reduction was observed (0.9 ± 1.3), reaching the best outcome at Week 208 (0.4 ± 0.8), in line with the trends seen in the overall EASI score.

**TABLE 2:** Mean baseline scores and effectiveness outcomes at Week (W) 16, 52, 104, 156, 208, and 260.

AD severity score *N*	Baseline value [SD] 583	16 W value [SD] 504	52 W value [SD] 408	104 W value [SD] 335	156 W value [SD] 230	208 W value [SD] 136	260 W value [SD] 59
EASI	23.5 [9.8]	4.1 [5.4]	3.0 [4.5]	1.8 [2.6]	1.6 [2.8]	1.4 [2.4]	1.6 [3.4]
EASI reduction (%)	—	82.7	87.3	92.3	93.0	93.9	93.0
EASI‐75 (%)	—	79.8	85.2	92.4	92.0	93.2	93.0
EASI‐90 (%)	—	55.4	60.5	71.2	74.7	78.9	80.7
EASI‐100 (%)	—	34.7	34.4	44.6	47.1	54.1	50.9
H&N EASI	3.3 [2.7]	0.9 [1.3]	0.8 [1.3]	0.6 [1.4]	0.5 [0.8]	0.4 [0.8]	0.4 [0.8]
H&N EASI reduction (%)	—	71.7	75.7	82.8	85.9	86.5	86.5
P‐NRS	8.5 [1.9]	3.2 [2.6]	2.9 [2.5]	2.3 [2.3]	1.9 [2.1]	1.7 [2.1]	1.9 [2.2]
P‐NRS reduction (%)	—	61.8	66.3	73.3	78.0	80.1	77.7
P‐NRS 0/1 (%)	1.1	29.4	35.3	46.5	52.2	60.5	56.9
MDA (%)	—	22.0	30.6	40.0	45.6	56.0	55.3
S‐NRS	7.2 [5.5]	1.1 [2.2]	0.5 [1.9]	0.5 [1.7]	0.3 [1.3]	0.3 [1.1]	0.3 [0.9]
S‐NRS reduction (%)	—	84.8	92.8	92.8	95.5	95.5	95.5
DLQI	14.5 [7.2]	4.7 [5.0]	3.7 [4.5]	3.0 [4.1]	2.1 [3.3]	2.0 [3.1]	1.7 [2.1]
DLQI reduction (%)	—	67.5	74.3	79.7	85.3	86.4	88.2

Abbreviations: AD, atopic dermatitis; DLQI, Dermatology Life Quality Index; EASI, Eczema Area and Severity Index; H&N, head and neck involvement; MDA, Minimal Disease Activity; P‐NRS, Pruritus‐Numerical Rating Scale; S‐NRS, Sleep Loss‐Numerical Rating Scale; SD, standard deviation.

EASI‐75, EASI‐90, and EASI‐100 were achieved by 79.8%, 55.4%, and 34.7% of patients, respectively, at Week 16. As with the mean EASI and H&N EASI scores, the best outcomes were observed at Week 208, with the only exception being EASI‐90, which showed its peak response at the final follow‐up at Week 260 (Table 2).

Similar results were obtained regarding sleep disturbance and itch control. The mean P‐NRS and S‐NRS values ± SD were 8.5 ± 1.9 and 7.2 ± 5.5 at baseline respectively. These values progressively decreased over the 5‐year follow‐up period, reaching their lowest levels after 3 years of treatment and remaining stable through the fifth year of therapy (Table 2).

The achievement of MDA demonstrated a favorable trend, increasing steadily throughout the study period. At Week 16, 22.0% of patients had achieved the outcome, with rates continuing to rise over time, attaining 56.0% at Week 208, and remaining stable through the 5‐year follow‐up (55.3%) (Table 2). Alongside the excellent results observed in PROs and clinical scores, a marked and rapid reduction in DLQI values was recorded, as shown in Table 2.

The results of the univariate analysis indicated that patients with infantile onset exhibited significantly higher EASI scores at Weeks 52 (*p* = 0.02) and 104 (*p* = 0.01), along with lower rates of MDA achievement at Weeks 52 (*p* = 0.01) and 260 (*p* = 0.01). Head and neck involvement was associated with reduced MDA achievement after 1 year of treatment (*p* = 0.02). Male patients showed higher EASI scores at Week 16 (*p* = 0.01) and higher baseline DLQI scores (*p* = 0.001). Additionally, greater pruritus intensity at Week 260 was observed in patients with infantile onset (*p* = 0.02). Prior cyclosporine use was associated with significantly higher head and neck EASI scores at baseline (*p* = 0.02) and Week 16 (*p* = 0.01), and with a reduced likelihood of achieving MDA at 3 years (*p* = 0.05). No significant associations were found for family history of atopy (Table 3).

**TABLE 3:** Univariate analysis of baseline variables and treatment outcomes over time.

Mean EASI	Baseline	16 weeks	52 weeks	104 weeks	156 weeks	208 weeks	256 weeks
Sex							
Male	24.12	4.65	3.15	1.92	1.96	1.65	1.79
Female	22.82	**3.43**	2.83	1.71	1.33	1.23	1.41
Infantile onset							
No	24.09	3.63	2.26	1.31	1.67	1.17	0.65
Yes	23.47	4.03	**3.28**	**2.07**	1.63	1.58	2.00
Family history of atopy							
No	23.48	4.22	2.74	1.82	1.47	1.41	2.00
Yes	2.50	3.64	3.25	1.69	1.60	1.61	1.35
Previous Ciclosporine							
No	22.95	4.56	2.85	1.87	2.26	2.13	1.17
Yes	23.86	3.91	3.10	1.82	1.54	1.35	1.65
Head & Neck involvement							
No	22.29	4.04	2.58	0.27	1.02	0.96	0.57
Yes	23.94	4.13	3.09	0.64	**1.78**	1.59	1.75

Mean DLQI	Baseline	16 weeks	52 weeks	104 weeks	156 weeks	208 weeks	256 weeks
Sex							
Male	13.54	4.79	3.41	2.57	1.82	2.98	1.11
Female	**15.67**	4.65	4.07	3.32	2.44	3.26	2.27
Infantile onset							
No	14.35	5.76	3.68	3.20	1.99	1.73	0.88
Yes	14.74	4.22	3.75	2.81	2.22	2.12	2.02
Family history of atopy							
No	14.47	5.05	3.82	2.93	1.86	2.12	1.25
Yes	15.10	4.40	3.79	2.77	2.25	1.88	2.30
Previous Ciclosporine							
No	13.51	4.96	3.17	2.78	2.70	3.94	1.20
Yes	14.98	4.66	3.94	3.00	2.06	1.73	1.75
Head & Neck involvement							
No	13.70	4.22	3.25	3.04	2.23	1.77	2.38
Yes	14.80	4.89	3.85	2.89	2.10	1.99	1.60

Mean NRSp	Baseline	16 weeks	52 weeks	104 weeks	156 weeks	208 weeks	256 weeks
Sex							
Male	8.31	3.30	2.69	2.19	1.86	1.72	1.38
Female	8.73	3.21	3.04	2.35	1.88	1.67	2.41
Infantile onset							
No	—	3.59	2.41	2.14	1.95	1.48	0.88
Yes	—	3.04	3.06	2.33	1.87	1.82	2.29
Family history of atopy							
No	8.60	3.39	2.86	2.13	1.91	1.69	1.46
Yes	8.48	2.97	2.90	2.41	1.74	1.84	2.44
Previous Ciclosporine							
No	8.48	3.70	2.96	2.23	2.55	2.00	1.20
Yes	8.60	3.10	2.87	2.29	1.77	1.67	1.96
Head & Neck involvement							
No	8.36	3.34	2.21	1.88	1.95	1.77	2.00
Yes	8.54	3.24	3.02	2.35	1.82	1.69	1.88

MDA %	Baseline	16 weeks	52 weeks	104 weeks	156 weeks	208 weeks	256 weeks
Sex							
Male		24.3%	29.8%	37.0%	44.0%	55.1%	53.6%
Female		19.8%	31.4%	43.3%	47.3%	56.9%	57.1%
Infantile onset							
No		18.9%	40.0%	48.0%	44.9%	59.1%	81.3%
Yes		24.6%	**25.70%**	36.7%	45.5%	53.9%	**45.0%**
Family history of atopy							
No		21.1%	27.80%	40.4%	43.4%	55.1%	64.3%
Yes		25.2%	32.50%	39.7%	53.0%	52.9%	44.0%
Previous Ciclosporin							
No		19.8%	26.80%	42.4%	29.0%	50.0%	60.0%
Yes		23.2%	30.80%	39.2%	**48.0%**	56.4%	54.9%
Head & Neck involvement							
No		21.0%	42.40%	50.9%	42.5%	63.6%	66.7%
Yes		22.7%	**27.50%**	37.0%	46.2%	53.7%	54.0%

*Note:* Bold indicates *p* < 0.05.

Abbreviations: DLQI, Dermatology Life Quality Index; EASI, Eczema Area and Severity Index; MDA, Minimal Disease Activity; P‐NRS, Pruritus‐Numerical Rating Scale; S‐NRS, Sleep Loss‐Numerical Rating Scale.

The multivariate analysis identified several factors associated with the likelihood of achieving MDA across different time points. Higher baseline EASI score was significantly associated with increased odds of achieving MDA at Week 16 (*p* = 0.03). Infantile onset was significantly associated with lower odds of achieving MDA at Week 52 (*p* = 0.02) and Week 260 (*p* = 0.05). Head and neck involvement was also associated with reduced odds of achieving MDA at Week 52 (*p* = 0.04) and showed a trend toward significance at Week 104 (*p* = 0.07). No other variables demonstrated statistically significant associations across the remaining time points (Table 4).

**TABLE 4:** Multivariate analysis of factors influencing the achievement of MDA across multiple time points.

	16 w OR (IC95%)	52 w OR (IC95%)	104 w OR (IC95%)	156 w OR (IC95%)	208 w OR (IC95%)	260 w OR (IC95%)
Sex (M vs. F)	0.79 (0.50–1.25)	1.05 (0.67–1.66)	1.31 (0.82–2.10)	1.07 (0.61–1.86)	1.01 (0.49–2.11)	0.83 (0.25–2.73)
Infantile onset	1.48 (0.88–2.48)	**0.56 (0.34–0.90)**	0.75 (0.45–1.25)	0.78 (0.43–1.42)	0.89 (0.40–1.99)	**0.24 (0.05–1.07)**
Family history of atopy	1.17 (0.72–1.88)	1.31 (0.80–2.14)	1.08 (0.66–1.76)	1.49 (0.83–2.68)	0.77 (0.36–1.65)	0.41 (0.12–1.40)
Previous Ciclosporine	1.19 (0.69–2.05)	1.17 (0.68–2.02)	0.91 (0.51–1.65)	2.13 (0.92–4.94)	1.33 (0.44–3.98)	0.99 (0.11–8.66)
EASI score T0	**1.03 (1.00–1.05)**	1.01 (0.99–1.03)	1.00 (0.98–1.02)	1.01 (0.98–1.03)	1.02 (0.99–1.06)	1.05 (0.98–1.11)
Head & Neck involvement	0.96 (0.55–1.68)	0.54 (0.31–0.96)	**0.58 (0.31–1.06)**	1.06 (0.51–2.19)	0.60 (0.22–1.62)	0.73 (0.10–5.59)

*Note:* Bold indicates *p* < 0.05.

Abbreviations: 95% CI, 95% confidence intervals; F, female; M, male; OR, odds Ratio; T0, baseline; W, week.

A total of 58 patients (9.9%) discontinued therapy, with 44 (7.5%) due to inefficacy and 12 (2.1%) due to adverse events. A total of 66 patients (11.3%) were reported lost to follow‐up, and 3 patients (0.5%) died from causes unrelated to dupilumab therapy. The most common adverse event reported was conjunctivitis, with a total of 137 cases (23.5%), followed by herpes infection (66 cases, 11.3%). All the adverse events are described in Table 5.

**TABLE 5:** Adverse events at each time point and overall.

Event	4 months	1 year	2 years	3 years	4 years	5 years	Total
*N*	504	408	335	230	136	59	583
Conjunctivitis	75	34	14	11	2	1	137
Herpes	31	15	12	5	3	0	66
Injection site reaction	6	1	0	0	0	0	7
Helminth infection	1	0	0	0	0	0	1
Headache	8	1	0	1	0	0	10
Alopecia	3	2	1	1	0	0	7
Asthenia	4	0	0	0	0	0	4
Arthralgia	2	1	4	0	0	1	8
Psoriasis	0	0	2	3	1	0	6

**FIGURE 1:**
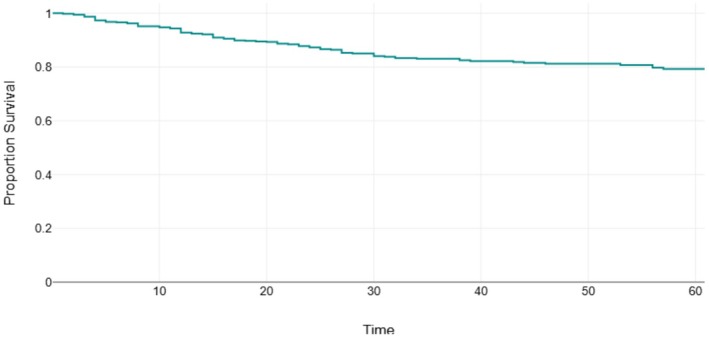
Drug survival of dupilumab through 260 weeks of treatment.

The drug survival rate was 79.3% after 5 years of treatment (Figure 1). A multivariate binary logistic regression analysis was performed to identify independent predictors of drug interruption, adjusting for potential confounders including sex, childhood onset, family history of atopy, prior cyclosporine use, baseline EASI score, and head and neck involvement. Sex emerged as a significant predictor of treatment discontinuation (*p* = 0.01), with males having 1.75 times higher odds of therapy interruption compared to females (Table 6).

**TABLE 6:** Multivariate logistic regression of predictors for drug interruption.

	Coefficients	Lower 95% CI	Upper 95% CI	Std. error	z	*p*	Exp(B)	Lower 95% CI	Upper 95% CI
Sex	0.56	0.13	0.99	0.22	2.54	**0.011**	1.75	1.14	2.68
Infantile onset	0.02	−0.42	0.46	0.23	0.1	0.923	1.02	0.66	1.59
Family history of atopy	−0.13	−0.58	0.33	0.23	0.54	0.588	0.88	0.56	1.39
Previous cyclosporine	−0.02	−0.53	0.48	0.26	0.1	0.923	0.98	0.59	1.62
EASI score T0	0	−0.02	0.02	0.01	0.37	0.714	1	0.98	1.02
H&N involvement	0.54	−0.08	1.16	0.32	1.71	0.088	1.72	0.92	3.20

*Note:* Bold indicates *p* < 0.05.

Abbreviations: 95% CI, confidence intervals; EASI, Eczema Area and Severity Index; H&N, head and neck; T0, baseline.

## 4 Discussion

Dupilumab is recognized as a highly effective and safe therapy for patients affected by moderate to severe AD both in pediatric and adult patients [12, 13, 14, 15]. While the safety and efficacy initially demonstrated in clinical trials have been confirmed by many short‐term real‐world studies, long‐term real‐life data remain limited [12, 16]. Moreover, these studies frequently assess clinical scores, such as EASI, and PROs independently, without considering a composite outcome that captures both clinical signs and patients' symptoms [11, 17]. In recent years, based on the excellent results achieved with new systemic therapies, treatment goals have become increasingly ambitious. In this context, the concept of MDA has emerged as a therapeutic target, defined as the achievement of an optimal balance between objective clinical improvement and PROs, rather than the mere absence of visible disease or symptoms [11, 17]. In our study, MDA was defined as the simultaneous achievement of EASI‐90 and a P‐NRS score ≤ 1. However, no universal definition exists, and other studies could include measures such as the DLQI or S‐NRS. This variability in criteria should be considered when comparing our findings with those of other cohorts, as the use of a more restricted definition may lead to differences in estimated MDA rates.

As far as we know, there are very limited real‐life data with a 5‐year follow‐up on patients with moderate‐to‐severe AD treated with dupilumab. In addition, no real‐world study has specifically investigated the achievement of MDA in this patient population.

An initial review of our results indicates that dupilumab significantly reduces disease burden beginning at Week 16 of treatment, with the clinical response sustained consistently over a 5‐year period (Table 2). These findings are closely aligned with those reported in the current literature [18, 19, 20]. Notably, our data show slightly improved outcomes compared to the 5‐year open‐label study, particularly in terms of lower mean EASI scores from Week 52 (3.0 vs. 3.2) through Week 260 (1.6 vs. 2.8) [19]. These results translated into higher rates of EASI‐75 and EASI‐90 achievement after 5 years of treatment, reaching 93.0% and 80.7%, respectively, compared to 88.9% and 76.2% reported in the clinical trial [18]. Outcomes related to itch control were comparable between the two studies, with only minimal, non‐significant differences observed [19].

We compared our results with a similar real‐life study conducted in Italy with a follow‐up of 5 years [20]. Our data on effectiveness outcomes appear slightly higher yet remain broadly comparable to those reported by Barei et al. [20]. Specifically, EASI and P‐NRS scores at year 5 were 1.6 versus 1.0 and 1.9 versus 1.0, respectively. When analyzing the percentage reduction in EASI scores, our data show a higher achievement of EASI‐100 at 4 and 5 years of treatment, with rates of 54.1% versus 46.9% and 50.9% versus 37.5%, respectively [20]. Comparable, albeit slightly lower, outcomes were also observed for EASI‐75 and EASI‐90 [20].

A different trend in effectiveness outcomes is observed when comparing our results with those from the multicenter Dutch studies based on the BioDay registry. In particular, the mean EASI and the proportion of patients achieving EASI‐90 after 5 years of therapy differ substantially between the two cohorts (2.9% vs. 1.6% and 46.2% vs. 80.7%, respectively). This discrepancy may stem from the markedly different sample sizes, as well as from the considerably higher proportion of patients undergoing tapering in the Dutch cohort compared with our cohort (70.5% vs. 16.3%) [21, 22]. Moreover, the Dutch research group adopted a different approach for assessing the achievement of MDA, defining it as the attainment of at least two of the following parameters: POEM ≤ 7, DLQI ≤ 5, and P‐NRS ≤ 4. This represents a less stringent outcome measure compared with the definition used in our study, making a meaningful comparison between the two cohorts not feasible [21].

Some interesting findings emerged from the analysis of potential predictors of treatment response. In particular, female sex appeared to influence the early treatment response, with lower mean EASI scores observed after the first 16 weeks of therapy (3.43 vs. 4.65, *p* = 0.01). Several real‐life studies have reported sex‐based differences in treatment outcomes [21, 23, 24, 25], a finding further supported by a recent systematic review and meta‐analysis, which identified female sex as a positive predictive factor for response to dupilumab therapy [26]. It is well known how immune response differs between the sexes and is influenced by various factors, among which the role of hormones assumes a central position [27]. These aspects could lead to differences in disease burden and response to therapies that interact with the immune system, such as dupilumab, among patients of different sexes [26, 27]. Similar results are observed regarding the impact of childhood onset on treatment response; in particular, patients with documented disease onset during childhood show higher mean EASI scores after 1 and 2 years of therapy. This phenomenon may be attributable to a longer disease duration, potentially indicative of a sustained underlying inflammatory state, thereby contributing to reduced therapeutic responsiveness and increased treatment resistance over time. Although recent evidence suggests that age of onset may not significantly influence the response to dupilumab therapy [26, 28]. Instead, the age at which treatment is initiated appears to be more relevant, with earlier initiation, particularly during childhood, being associated with more favorable clinical outcomes [25, 28].

Regarding the achievement of MDA, our study demonstrates favorable outcomes, with a progressive increase beginning at the first follow‐up at 16 weeks (22.0%) and continuing steadily through 4 years of treatment (56.0%), eventually reaching an apparent plateau that is maintained through 5 years (55.3%). Baseline characteristics, such as head and neck involvement, childhood‐onset disease, and baseline EASI score, appear to influence the achievement of MDA. Although the associations observed in our data are sporadic and not consistently maintained across different timepoints, resulting in a rather uncertain interpretation.

Despite the positive results described, MDA rates remain lower compared to individual effectiveness outcomes (Table 2), underscoring the importance of adopting MDA as a primary therapeutic goal. This approach emphasizes the need to consider not only the clinical presentation but also the patient's subjective symptom experience in the overall assessment of treatment success [10, 11].

Our experience confirms the well‐known safety of dupilumab [19, 20, 21, 29, 30, 31, 32], including its long‐term tolerability, with a low incidence of adverse events, the majority of which were of mild or minimal severity. Indeed, only 12 (2.1%) patients in our cohort discontinued therapy due to adverse events.

Conjunctivitis was the most frequently reported adverse event associated with dupilumab therapy. Although it is a well‐recognized and typically manageable side effect, it remains the most common reason for treatment interruption due to adverse events, though it rarely leads to permanent discontinuation [30, 33].

Dupilumab demonstrated excellent drug survival, with 79.3% of patients remaining on treatment after 5 years. Most discontinuations were due to lack of efficacy, further highlighting its excellent safety profile. These results are consistent with findings from the multicentric Italian study conducted over a 5‐year period [31]. Comparable drug survival data also emerge from the multicentric studies conducted in the Netherlands using the BioDay registry; however, these cohorts show a different trend, with adverse events, rather than lack of efficacy, representing the leading cause of treatment discontinuation [22, 34]. Similar to effectiveness outcomes, male sex also appears to have a negative impact on drug survival, being associated with a higher rate of treatment discontinuation compared to female sex. Although several studies have described the role of sex in relation to dupilumab therapy, its correlation remains unclear. Further in‐depth studies are needed to better understand this aspect [23, 24, 26].

The limitations of this study include its retrospective and monocentric design, the small sample size at the later follow‐ups, and the potential positive selection bias introduced by the discontinuation of patients with inadequate response over time. Furthermore, statistical modeling approaches such as linear mixed models were not used, which may limit the ability to fully account for missing data, repeated measures, and individual variability. Although dupilumab appears to remain effective in the long term, conclusions regarding further improvements at later time points should be interpreted with caution. In addition, information on concomitant systemic or topical therapies during dupilumab treatment was not consistently available, limiting the possibility to assess their impact on outcomes.

## 5 Conclusion

Our data add to the long‐term evidence supporting the effectiveness and safety of dupilumab in patients with moderate‐to‐severe AD, demonstrating consistent and sustained outcomes that align with findings from both clinical trials and real‐world experiences reported in the current scientific literature [18, 19, 20, 26, 35, 36, 37].

As far as we know, this is the first long‐term real‐life study assessing the achievement of MDA in patients treated with dupilumab for moderate‐to‐severe AD. The results are promising, with more than 55% of patients achieving the outcome after 4 years and maintaining this outcome through 5 years, highlighting the sustained and progressive benefits of long‐term dupilumab therapy. Male sex and childhood‐onset appear to negatively impact effectiveness outcomes and drug survival, underscoring the need for further investigation and validation of these findings in real‐world, long‐term studies.

## Funding

The authors have nothing to report.

## Ethics Statement

The study was conducted in accordance with the Declaration of Helsinki and approved by the Ethics Committee of the Department of Medicine at the University of Turin.

## Consent

Informed consent was obtained from all subjects and parents involved in the study.

## Conflicts of Interest

The authors declare no conflicts of interest.

## Data Availability

The data that support the findings of this study are available on request from the corresponding author. The data are not publicly available due to privacy or ethical restrictions.
